# Individual or combinational use of phytase, protease, and xylanase for the impacts on total tract digestibility of corn, soybean meal, and distillers dried grains with soluble fed to pigs

**DOI:** 10.5713/ab.23.0212

**Published:** 2023-08-28

**Authors:** Adsos Adami Passos, Vitor Hugo Cardoso Moita, Sung Woo Kim

**Affiliations:** 1 Department of Animal Science, North Carolina State University, Raleigh, NC 27695, USA

**Keywords:** Corn, Distillers Dried Grains with Soluble (DDGS), Phytase, Pigs, Protease, Soybean Meal, Xylanase

## Abstract

**Objective:**

This study was to evaluate the effects of individual or combinational use of phytase, protease, and xylanase on total tract digestibility of corn, soybean meal, and distillers dried grains with soluble (DDGS) fed to pigs.

**Methods:**

Each experiment had four 4×4 Latin squares using 16 barrows. Each period had 5-d adaptation and 3-d collection. All experiments had: CON (no enzyme); Phy (CON+phytase); Xyl (CON+xylanase); Pro (CON+protease); Phy+Xyl; Phy+Pro, Xyl+Pro, Phy+Xyl+Pro. Each Latin square had ‘CON, Phy, Xyl, and Phy+Xyl’; ‘CON, Phy, Pro, and Phy+Pro’; ‘CON, Pro, Xyl, and Xyl+Pro’; and ‘Phy+Xyl, Phy+Pro, Xyl+Pro, Phy+Xyl+Pro’.

**Results:**

The digestible energy (DE), metabolizable energy (ME), and nitrogen retention (NR) of corn were not affected by enzymes but the apparent total tract digestibility (ATTD) of phosphorus (P) was improved (p<0.01) by Phy. The DE and ATTD dry matter (DM) in soybean meal were increased (p<0.05) by Phy+Pro and the ATTD P was improved (p<0.01) by Phy, Phy+Pro, and Phy+Xyl. The DE, ME, and ATTD DM in DDGS were improved (p<0.05) by Phy+Xyl and the ATTD P was improved (p<0.01) by Phy, Phy+Pro, and Phy+Xyl.

**Conclusion:**

Phytase individually or in combination with xylanase and protease improved the Ca and P digestibility of corn, soybean meal, and DDGS, from the hydrolysis of phytic acid. The supplementation of protease was more effective when combined with phytase and xylanase in the soybean meal and DDGS possibly due to a higher protein content in these feedstuffs. Xylanase was more effective in DDGS diets due to the elevated levels of non-starch polysaccharides in these feedstuffs. However, when xylanase was combined with phytase, it demonstrated a higher efficacy improving the nutrient digestibility of pigs. Overall, combinational uses of feed enzymes can be more efficient for nutrient utilization in soybean meal and DDGS than single enzymes.

## INTRODUCTION

Corn, soybean meal, and distillers dried grains with soluble (DDGS) are commonly used feedstuffs in pig production, providing energy, protein, and other essential nutrients to pigs [1,2]. Although these feedstuffs are highly digestible providing nutrients, they contain undigestible components and antinutritional factors that can negatively impact the health and growth performance of pigs [3,4]. Phytic acid, non-starch polysaccharides (NSP), allergenic proteins, and trypsin inhibitors are some of the main antinutritional factors that can be found in corn, soybean meal, and DDGS [5–8].

To mitigate some of the negative effects of these undigestible components and antinutritional factors, the supplementation of feed enzymes, individually or combined, can be an effective strategy to break down specific components in the feed and consequently improve nutrient digestibility, intestinal health and growth performance [9–12]. Xylanase breaks down the β-1,4 xylan bonds present in plant-cell walls enhancing nutrient digestibility, intestinal health, and reducing the negative impacts associated with NSP [13–16]. Phytase catalyzes the hydrolysis of phytic acid reducing its biding capacity and increasing bone and intestinal health, and the bioavailability of essential minerals and nutrients [17–19]. Proteases are enzymes that can break down proteins (including glycinin and conglycinin), increasing their digestibility and bioavailability that can improve growth performance of the animals [20–22].

The combination of selected feed enzymes has shown potential benefits on nutrient digestibility, intestinal health, and growth performance of pigs [1,2,23]. Combining phytase, protease, and xylanase in pig diets is proposed to bring synergistic effects from the elimination of anti-nutritional compounds for the enhanced intestinal health and improved nutrient utilization. The combined use of phytase and xylanase enhanced nutrient utilization from the hydrolysis of phytates and improved intestinal health from oligosaccharides hydrolyzed from xylans and arabinoxylans in pigs [24–26]. The combined use of protease with xylanase and phytase increased the destruction of allergenic soy proteins and improved nutrient utilization [27–29]. In addition to their nutritional benefits, combining these enzymes can potentially reduce feed costs and environmental concerns regarding the manure composition [28,30].

Therefore, it was hypothesized that the use of selected combinations of feed enzymes can effectively break down anti-nutritional compounds and enhance the nutrient digestibility of some of the main feedstuffs used in diets for pigs. To test the hypothesis, three experiments were conducted to evaluate the use of phytase, protease, and xylanase individually or in all possible combinations on nutrient digestibility of corn, DDGS, and soybean meal fed to pigs.

## MATERIALS AND METHODS

The three experiments were conducted at the Swine Educational Unit of North Carolina State University (Raleigh, NC, USA). The experimental protocol was approved by the Institutional Animal Care and Use Committee of North Carolina State University.

### Feedstuffs and feed enzymes

Feedstuffs used in the experiments were corn (IFN 4-02-861), soybean meal (IFN 5-04-612), and DDGS (IFN 5-02-843). Particle size of the ingredients was measured according to ASAE [31]. The geometric mean diameter (Dgw) of the corn particles was 451 μm. The standard deviation of geometric mean diameter (Sgw) was 3.9. The Dgw of the soybean meal particles was 872 μm and Sgw was 2.5. The Dgw of the DDGS particles was 969 μm and Sgw was 2.0.

Feed enzymes used in the experiments were phytase (myo-inositol hexakiphosphate phosphohydrolase, Ronozyme HiPhos; DSM, Parsippany, NJ, USA), protease (serine endopeptidase, Ronozyme ProAct; DSM, USA), and xylanase (endo-1,4-β-xylanase, Ronozyme WX; DSM, USA).

### Animals, design, and diets

Three experiments were conducted to evaluate total tract digestibility of nutrients in corn, soybean meal, and DDGS fed to pigs. The experiment 1 was conducted to evaluate the nutrient digestibility of corn using 16 pigs with initial body weight (BW) of 39.2±2.4 kg. A diet with corn (Table 1) was formulated to contain 95% of corn (Table 2). The experiment 2 was conducted to evaluate the nutrient digestibility of soybean meal using 16 pigs (initial BW of 23.5±4.1 kg). A diet with soybean meal (Table 1) was formulated to contain 26% of soybean meal (Table 2) and 71.5% of corn (same corn used in experiment 1). The experiment 3 was conducted to evaluate the nutrient digestibility of DDGS using 16 pigs (initial BW of 24.0±3.7 kg). A diet with DDGS (Table 1) was formulated to contain 35% of DDGS (Table 1) and 61.6% of corn (same corn used in experiment 1). The dietary treatments were: CON (no enzyme); Phy (CON+phytase); Xyl (CON+xylanase); Pro (CON+protease); Phy+Xyl; Phy+Pro, Xyl+Pro, Phy+Xyl+Pro. Phytase inclusion was 1,000 FTU/kg of feed where one FTU was defined as the amount of phytase needed for the release of 1 μmol of inorganic P per minute from an excess of 15 M sodium phytate at pH 5.5 and 37°C [32]. Protease inclusion was 15,000 PRO/kg of feed where one PRO is defined as the amount of protease that liberates 1 μmol para-nitroaniline from 1 mM Suc-Ala-Ala-Pro-PhepNA substrate per minute at pH = 9.0 and at 37°C [33]. Xylanase inclusion was 200 FXU/kg of feed where one FXU unit is defined as the amount of xylanase that liberates 7.8 μmol of reducing sugars/min from azo-wheat arabinoxylan at pH 6.0 and 50°C [34].

Each experiment was composed of four 4×4 Latin squares, 16 barrows of each experiment were randomly assigned to each of the four Latin squares in the same room and were evaluated during four periods. Genetic backgrounds of pigs were originated from Smithfield Premium Genetics, and all internally bred at Swine Educational Unit of North Carolina State University (Raleigh, NC, USA). Each period consisted of 8 d (5 d adaptation and 3 d collection). Each Latin square had ‘CON, Phy, Xyl, and Phy+Xyl’; ‘CON, Phy, Pro, and Phy+ Pro’; ‘CON, Pro, Xyl, and Xyl+Pro’; and ‘Phy+Xyl, Phy+Pro, Xyl+Pro, Phy+Xyl+Pro’, respectively. Representative samples from each experimental diets were collected as diets were mixed and stored at −20°C for further analysis. Pigs received experimental diets twice daily (07:00 and 17:00 h) at a fixed amount based on BW of pigs (daily feed allowance = 0.09× BW^0.75^). Pigs were weighed at the end of each collection period to adjust feed allowance for a subsequent period. Daily feed intake was recorded considering any feed refusal.

### Sample collection, processing, and analysis

On the last day of each adaption period at 17:00 h, chromium oxide (0.5%) was added to the evening meal as an external marker indicating the start of fecal collection. Sampling procedures were done for three consecutive days. Fecal collection was initiated when green color from chromium oxide was observed in the feces in the following day, whereas urine sampling was initiated from the time of feeding a meal with chromium oxide.

On the last day of each collection period at 17:00 h, chromium oxide (0.5%) was added to the evening meal as an external marker indicating the end of fecal collection. Fecal sampling was ceased when green color was observed in the feces in the following day Urine collection was ceased at the time of evening meal on the last day of collection period. Urine samples were collected in a plastic bucket with 20 mL concentrated HCl (5 *N*). Volume of urine was measured each day during the collection period and 150 mL of urine sample was daily sub-sampled. Fecal samples were weighed at the end of each day during the collection period. Urine and fecal samples were frozen (−20°C) immediately after collection.

Fecal samples were oven dried in forced-air oven at 65°C. Fecal and feed samples were analyzed for moisture (Method 934.01 [35]). Nitrogen was determined by combustion method (FP528; Leco, St Joseph, MI, USA) to calculate crude protein (CP) (Method 992.15 [32]). Gross energy (GE) was determined using adiabatic bomb calorimeter (C2000; IKA, Wilmington, NC, USA). Total ash (Method 942.05 [32]), acid detergent fiber (Method 973.18), neutral detergent fiber (2002.04), and ether extract (Method 2003.06), Ca (Method 968.08 [32]), and P (method 946.06 [32]) were also analyzed for the experiments done with corn, soybean meal, and DDGS. Urine samples were freeze-dried (24D x 48; Virtis, Gardiner, NY, USA) and analyzed for N and GE as previously described.

### Apparent total tract digestibility and nitrogen retention of feedstuffs and diets

Total feed intake for each animal of both experiments was summarized at the conclusion of each experiment. Experiment 1 considered corn as a test ingredient. Experiments 2 and 3 considered soybean meal and DDGS as test ingredients, respectively, whilst energy and nutrient digestibility from corn were obtained from the experiment 1. After analysis, the percentage of nutrient contribution of basal ingredients and test ingredients in the test diets were calculated [36,37]. The laboratory results of dry matter (DM), GE, N, ash content, Ca, and P were utilized for the calculations of digestible energy (DE), metabolizable energy (ME), nitrogen retention (NR), and apparent total tract digestibility (ATTD) of DM, N, ash, Ca, and P. Total feed intake for each animal was summarized at the conclusion of each experiment. For ATTD, the intake of DM, ash, Ca, P, and N were then calculated for each animal by multiplying feed intake by the analyzed concentration of each nutrient in the diets. The excretions of DM, ash, Ca, P, and N in feces were calculated for each pig by multiplying the analyzed concentrations by the total quantity of feces excreted by each pig. The ATTD of each nutrient was calculated according to following equation as previously described by Stein et al [38]:


ATTD of nutrient (%)=[(Ni-Nf)/Ni]×100

Where ATTD is the apparent total tract digestibility of nutrients; Ni is the total intake of nutrients during the three days of collection period; and Nf is the total fecal output of nutrients originating from the feed that was fed during the three days of collection period.

For NR, the total feed intake for each animal was summed up at the conclusion of each experiment. The intake of N was then calculated for each animal by multiplying feed intake by the analyzed concentration of each nutrient in the diets. The excretion of N in urine and feces were calculated for each pig by multiplying the analyzed concentrations by the total quantity of feces excreted by each pig. The NR as a percentage of intake was calculated according to the following equation as previously described by Stein et al [38]:


NR (%)=(Ni-(Nf+Nu)×100)/Ni

Where NR is total nitrogen retention; Ni is the nitrogen intake in the diets during the three days of collection period; Nf is the nitrogen amount in feces during the three days of collection period; and Nu is the nitrogen amount in urine during the three days of collection period.

### Statistical analysis

The data from experiments 1, 2, and 3 were analyzed using the GLIMMIX procedure of SAS 9.3 (SAS Inst. Inc., Cary, NC, USA). In each experiment, Latin square and period were included as fixed effects. Pig nested to Latin square was included as a random effect. Each of the four individual pigs randomly assigned to the four Latin Squares was the experimental unit. When the interaction was significant, multiple comparisons were performed using the least significant difference procedure with Tukey’s adjustment. Statistical differences were considered significant with p<0.05 and tendency with 0.05≤p<0.10.

## RESULTS

The feed enzymes supplemented to the diets were analyzed in the feed samples. The averaged results were 1,073 FTU/kg feed for the diets supplemented with phytase, 16,635 PRO/kg feed for the diets supplemented with protease, and 146 FXU/kg feed for the diets supplemented with xylanase.

### Experiment 1

No effects of feed enzymes were detected on the ATTD of DM and N, DE, ME, and NR in the corn and corn diets when compared with CON (Table 3). The supplementation of Phy and Phy+Pro improved the ATTD of ash in pigs fed corn and corn diets when compared with CON. The supplementation of Phy+Pro, and Phy+Xyl+Pro improved the ATTD of Ca in pigs fed corn and corn diets when compared with CON. The supplementation of Phy, Phy+Pro, Phy+Xyl, and Phy+Xyl+Pro improved the ATTD of P in pigs fed corn and corn diets when compared with CON.

### Experiment 2

No effects of feed enzymes were detected on the ME and NR in the soybean meal and soybean meal diets when compared with CON (Table 4). The supplementation of Pro, Phy+Pro, and Phy+Xyl improved the ATTD of DM and DE in the soybean meal and soybean meal diets when compared with CON.

The supplementation of Pro improved the ATTD of N in pigs fed soybean meal diets when compared with Xyl+Pro, whereas the supplementation of Pro and Phy+Pro improved the ATTD of N in pigs fed soybean meal when compared with Phy and Xyl+Pro. The supplementation of Phy+Pro and Phy+Xyl improved the ATTD of ash in pigs fed soybean meal and soybean meal diets when compared with CON. The supplementation of Phy, Phy+Pro, and Phy+Xyl improved the ATTD of Ca in pigs fed soybean meal and soybean meal diets when compared with CON. The supplementation of Phy, Phy+Pro, and Phy+Xyl improved the ATTD of P in pigs soybean meal diets when compared with CON, whereas only Phy tended to improve the ATTD of P in pigs fed soybean meal when compared with CON.

### Experiment 3

Nutrient digestibility in DDGS was calculated from the nutrient digestibility in the DDGS diets after discounting the nutrient contribution from corn, supplemental amino acids, monocalcium phosphate, and limestone (Table 5). The supplementation of Pro, Xyl, and Phy+Xyl improved the ATTD of DM and DE in pigs fed DDGS and DDGS diets when compared with CON. The supplementation of Phy, Xyl, and Phy+Xyl improved ME and ATTD of N in pigs fed DDGS and DDGS diets when compared with CON.

The supplementation of Pro tended to improve the NR in pigs fed DDGS and DDGS diets when compared with Phy, Xyl+Pro, and Phy+Xyl+Pro. The supplementation of Phy, Pro, Xyl, Phy+Pro, and Phy+Xyl improved the ATTD of ash, Ca, and P in pigs fed DDGS and DDGS diets when compared with CON.

## DISCUSSION

### Experiment 1

Corn is a feedstuff containing highly digestible nutrients for pigs [39]. On the other hand, due to a high composition of starch, corn can contain relatively low amounts of protein and NSPs (Table 2). Corn-based diets are often low in essential minerals such as Ca and P, and the availability of these minerals can be further reduced by the presence of phytate [40]. This could lead to an unbalanced mineral status in pigs and negatively impact their growth and health.

The results of the present study showed that phytase supplemented individually or combined with xylanase and protease increased the ATTD of Ca, P, and ash, whereas xylanase and protease showed most of the benefits to nutrient digestibility when supplemented together and/or in combination with phytase. The positive effects of phytase supplementation for pigs, especially in Ca and P digestibility, has been showing consistency over the last decades [19,41,42]. On the other hand, the supplementation of xylanase and protease showed more variable results when compared to phytase, even when combined with other enzymes [21,27,29]. One of plausible reasons for this results variability regarding protease supplementation is that this enzyme may digest or reduce optimal activity of other enzymes during the enzyme product preparation and digestion process of the animals [22]. The number of studies investigating the effects of xylanase supplementation for pigs increased the last few years where authors started to investigate different mechanisms and properties of this enzyme [13,16,43]. When xylanase activity is debated, it is important to formulate the diets in order to provide the appropriate amount of the targeted substrate [13,15,16].

The results of the present study reaffirmed the positive effects of phytase supplementation in the Ca and P digestibility and showed that when combined with xylanase also improved the digestibility of these minerals in pigs fed corn-based diets. This suggests that the use of phytase individually or combined with xylanase and protease can be an alternative strategy for improving the mineral digestibility of pigs fed corn-based diets.

### Experiment 2

Soybean meal is a commonly used ingredient in animal feed, providing essential nutrients for pigs, especially proteins and AA. However, soybean meal may contain different antinutritional factors and allergenic proteins that can impair the nutrient digestibility and growth in pigs mainly in their earlier stages of life [44]. In accordance with the results reported in the experiment 1, the supplementation of phytase improved the ATTD of Ca and P when supplemented individually or in combination with xylanase or protease, once again showing a consistent effectiveness of phytase in the nutrient digestibility, especially Ca and P, in pigs. These findings suggests that the effectiveness of phytase may not be dramatically affected by protease [22]. Conversely, phytase could only improve DE and ATTD of DM and ash when combined with protease and xylanase.

The supplementation of protease in turn improved DE and ATTD when supplemented individually and in combination phytase. Chen et al [45] investigated the effects of protease supplemented individually for 6-week-old pigs. The authors noted a decrease in malondialdehyde concentration in both the small intestine and serum, alongside an enhancement in the digestibility of CP. However, protease seems to be more effective when supplemented in combination with other enzymes [23,29,46]. This may be due the possibility of protease affects other enzymes activity during the enzyme product preparation and digestion process of the animals [22]. Although the individual supplementation of xylanase showed positive effects in the DDGS diet [16,43,47], most of the benefits in the corn and soybean meal diets were observed when combined with other enzymes.

In conclusion, the supplementation of phytase individually or in combination xylanase and protease can effectively increase the ATTD of Ca and P in pigs fed soybean meal diets. Additionally, the result of the present study suggests that the combination use of feed enzymes in soybean meal diets can be a strategy to enhance nutrient digestibility and utilization due to an active hydrolysis of the antinutritional factors and allergenic proteins presents in this feedstuff.

### Experiment 3

In this study, the supplementation of phytase individually or in combination with xylanase and protease showed once again an efficacy improving the digestibility of Ca, P, and ash in pigs fed DDGS diets. The digestibility of P, Ca, and ash can be influenced by various factors, including diet composition, feed processing methods, and the presence of antinutritional factors or feed enzymes in the feed [1,25,38]. While there is a relationship between the digestibility of P and Ca due to their interplay in bone formation and mineralization [17,41], the relationship between their digestibility and the digestibility of ash is not always correlated. Studies regarding the effect of dietary supplementation of phytase on P digestibility in DDGS are variable. Yáñez et al [26] reported a 10% improvement in ATTD of P due to dietary phytase supplementation. Almeida and Stein [48] reported a marginal 6% improvement in ATTD of P compared with 6.4% from the present study. Although the objective of our study was not to compare differences among ingredients, the data indicated that the improvement on ATTD of P of single phytase supplementation was 9.9% on DDGS, 53.0% on corn, and 12.4% on soybean meal. The phytate P represents 35% of the total phosphorus in DDGS whereas it is 80% in corn and 54% in soybean meal [39]. Process of corn fermentation in ethanol production reduces phytate concentration [49,50] and it could partially explain a reduced efficacy of phytase in DDGS diets.

Interestingly, this study also showed effects of the supplementation of xylanase and protease individually improving the ATTD of P. This may be due to release of non-phytate P trapped in undigested proteins and xylans and arabinoxylans by the action of protease and xylanase [5]. McAlpine et al [27] showed that the supplementation of protease increased protein digestibility in diets including wheat distillers grain fed to pigs. Interestingly, similar to experiment 2, the combinational use of protease with xylanase was not effective increasing nutrient digestibility in DDGS diets which is supported by previous studies [27,51]. This may be due to potential interaction between protease and other NSP enzymes [22] and high amount of NSP in DDGS and soybean meal in comparison to the corn.

In this study, xylanase showed a lower efficacy in diets containing DDGS compared to other studies in the literature [13,15,29]. This could be a result due to arabinofuranosyl side units attached to xylopyranosyl backbone of the arabinoxylan in corn [52], which was also included in the diet, and it can block the access of xylanase to xylopyranosyl backbone of the DDGS [53–55]. However, there is evidence that arabinofuranosyl groups attached to xylopyranosyl backbone of corn can be partially released under acidic pH conditions as in the stomach [56], thus enhancing the accessibility of xylanase to xylopyranosyl backbone for its degradation [57]. The fat content of DDGS is higher than corn [39], which may slow the gastric emptying [58,59] and in turn might favor enzymes with optimal activity enhanced in acidity environment, such as xylanase and phytase [60].

The supplementation of different feed enzymes has been showing a wide range of benefits for pigs at different stages of growth [13,16,43]. Additionally, research has demonstrated that the use of feed enzymes can lead to a reduction in fecal output, resulting in a more environmentally sustainable pig production system [28,61]. The mechanisms behind these benefits can vary depending on the diet composition, stage of growth of the pig, and type and inclusion level of the specific enzyme. Nevertheless, they all aim to hydrolyze or inactive antinutritional compounds, such as NSP and phytate, into more digestible forms, thus increasing the availability and utilization of nutrients by the pig.

## CONCLUSION

The supplementation of phytase individually or in combination with xylanase and protease improved the Ca and P digestibility of pigs fed diets with corn, soybean meal, and DDGS, reaffirming the efficacy of this enzyme over the hydrolysis of phytic acid. The supplementation of protease showed more effective when combined with phytase and xylanase in the soybean meal and DDGS diets possibly due to a higher protein content in these feedstuffs. Moreover, the supplementation of xylanase indicated to be more effective in DDGS diets due to the elevated levels of NSP in these feedstuffs. However, when xylanase was combined with phytase, it demonstrated a higher efficacy improving the nutrient digestibility of pigs. Overall, combinational uses of feed enzymes can be more efficient in improving nutrient utilization in soybean meal and DDGS fed to pigs compared with the use of single enzymes.

## Figures and Tables

**Table 1:** Ingredient composition, calculated composition, and analyzed composition (as-fed basis) of the diets

Item	Corn diet	Soybean meal diet	DDGS diet
Ingredient (%)			
Corn, yellow dent	95.24	71.50	61.60
Soybean meal dehulled, 48% CP	-	26.00	-
DDGS	-	-	35.00
L-Lys HCl	0.85	0.05	0.79
DL-Met	0.19	-	0.05
L-Thr	0.33	-	0.22
L-Trp	0.12	-	0.08
L-Val	0.27	-	0.08
L-Ile	0.24	-	0.07
Limestone	1.10	1.05	1.35
Monocalcium phosphate	0.90	0.64	-
Salt	0.40	0.40	0.40
Vitamin premix^1)^	0.06	0.06	0.06
Trace mineral premix^2)^	0.30	0.30	0.30
Calculated composition			
ME (Mcal/kg)	3.3	3.3	3.2
CP (%)	9.7	18.3	16.3
SID Lys (%)	0.83	0.83	0.83
SID Met + Cys (%)	0.47	0.52	0.47
SID Thr (%)	0.52	0.52	0.53
SID Trp (%)	0.15	0.16	0.15
SID Val (%)	0.56	0.70	0.56
SID Ile (%)	0.45	0.63	0.45
Ca (%)	0.60	0.60	0.60
P total (%)	0.46	0.50	0.45
P available (%)	0.23	0.23	0.24
Analyzed composition			
DM (%)	90.8	93.2	93.2
GE (kcal/kg)	3,825	3,832	3,960
CP (%)	9.5	18.4	16.6
EE (%)	2.4	1.6	4.1
NDF (%)	8.2	6.0	15.5
ADF (%)	3.0	2.9	6.9
Total ash (%)	3.0	5.2	4.3
Ca (%)	0.60	0.64	0.59
P (%)	0.37	0.45	0.46

DDGS, distillers dried grains with soluble; CP, crude protein; ME, metabolizable energy; SID, standardized ileal digestibility; Ca, calcium; P, phosphorus; DM, dry matter; GE, gross energy; EE, ether extract; NDF, neutral detergent fiber; ADF, acid detergent fiber.

1) Vitamin premix supplied per kg of feed: 12,341 IU of vitamin A as vitamin A acetate, 1,759 IU of vitamin D as cholecalciferol, 70.50 IU of vitamin E as tocopheryl acetate, 0.04 mg/kg of vitamin B12 as cyanocobalamin; 0.35 mg/kg of biotin, 5.82 mg/kg of vitamin K as menadione sodium bisulfite; 8.80 mg/kg of riboflavin, 35.27 mg/kg of pantothenic acid as calcium pantothenate; niacin, 52.91 mg/kg of niacin as nicotinamide, 2.65 mg/kg of folate as folic acid.

2) Trace mineral premix supplied per kg of feed: 33 mg/kg of Cu as copper sulfate, 331 mg/kg of Fe as ferrous sulfate, 79 mg/kg of Mn as manganous oxide, 330 mg/kg of Zn as zinc sulfate, 0.59 mg/kg of I as ethylenediamine dihydroiodide, 0.60 mg/kg of Se as sodium selenite.

**Table 2:** Analyzed composition of feedstuffs (as-is basis)

Item	Corn	Soybean meal	DDGS
DM (%)	90.2	91.4	94.2
GE (kcal/kg)	3,855	4,057	4,550
CP (%)	7.25	46.82	28.06
NDF (%)	9.07	6.36	28.53
ADF (%)	2.99	5.13	13.61
Total ash (%)	1.09	6.25	6.20
Ca (%)	0.01	0.25	0.02
EE (%)	2.57	0.74	7.50
P (%)	0.22	0.67	0.91

DDGS, distillers dried grains with soluble; DM, dry matter; GE, gross energy; CP, crude protein; NDF, neutral detergent fiber; ADF, acid detergent fiber; Ca, calcium; EE, ether extract; P, phosphorus.

**Table 3:** Apparent total tract digestibility (ATTD) of nutrients, and nitrogen retention (NR) of corn in pigs^1)^ fed corn diets with and without selected feed enzymes supplemented individually or combined

Item^2)^	Treatment^3)^								SEM	p-value

	CON	Phy	Pro	Xyl	Phy+Pro	Phy+Xyl	Xyl+Pro	Phy+Xyl+Pro		
Corn diet										
ATTD of DM (%)	90.6	91.0	91.8	95.0	91.2	90.9	90.4	90.2	0.4	0.517
DE (kcal/kg)	3,366	3,379	3,383	3,373	3,391	3,374	3,359	3,341	18	0.646
ME (kcal/kg)	3,307	3,302	3,322	3,314	3,324	3,303	3,294	3,270	18	0.566
ATTD of N (%)	83.2	83.7	84.2	83.5	84.7	84.1	83.6	83.8	0.8	0.776
NR (%)	68.7	68.4	69.4	68.3	69.6	66.7	68.4	68.8	1.2	0.731
ATTD of ash (%)	65.5^c^	70.0^ab^	66.6^bc^	64.4^c^	70.9^a^	67.9^abc^	65.1^c^	68.2^abc^	1.6	0.011
ATTD of Ca (%)	75.8^bc^	79.0^a^	77.1^ab^	73.8^c^	79.9^a^	75.7^bc^	74.7^bc^	80.8^a^	1.7	<0.001
ATTD of P (%)	60.7^d^	69.0^ab^	62.2^cd^	59.8^d^	71.0^a^	66.2^bc^	59.8^d^	67.0^abc^	2.1	<0.001
Corn										
ATTD of DM (%)	89.6	90.0	89.8	89.5	90.3	89.9	89.4	89.1	0.4	0.511
DE (kcal/kg)	3,316	3,328	3,334	3,325	3,341	3,323	3,309	3,291	18	0.657
ME (kcal/kg)	3,263	3,257	3,279	3,274	3,280	3,258	3,250	3,224	18	0.548
ATTD of N (%)	81.3	81.4	82.3	81.2	82.7	82.1	81.4	81.7	0.9	0.783
NR (%)	64.3	64.0	65.1	64.0	65.4	61.7	64.2	64.3	1.4	0.688
ATTD of ash (%)	56.8^c^	60.7^ab^	57.8^bc^	55.8^c^	61.5^a^	58.9^abc^	56.4^c^	59.1^abc^	1,4	0.010
ATTD of Ca (%)	43.9^bc^	45.7^ab^	44.8^abc^	43.3^c^	46.6^a^	44.5^bc^	43.6^bc^	47.2^a^	0,9	0.010
ATTD of P (%)	28.5^d^	43.6^ab^	31.3^cd^	27.0^d^	47.3^a^	38.5^bc^	26.9^d^	40.0^abc^	3.8	<0.001

SEM, standard error of the mean; ATTD of DM, apparent total tract digestibility of dry matter; DE, digestible energy; ME, metabolizable energy; ATTD of N, apparent total tract digestibility of nitrogen; NR, nitrogen retention; ATTD of ash. apparent total tract digestibility of ash; ATTD of Ca, apparent total tract digestibility of calcium; ATTD of P, apparent total tract digestibility of phosphorus.

1) Each least squares mean represents 4×4 Latin square replicated pens; 16 pigs were used in this experiment.

2) The values were calculated as dry matter basis.

3) CON, control treatment with no enzyme supplementation; Phy, Ronozyme Hiphos (DSM, Parsippany, NJ, USA), 100 mg/kg (1,000 FTU/kg feed); Pro, Ronozyme WX (DSM, Parsippany, NJ, USA), 200 mg/kg (200 FXU/kg feed); Xyl, Ronozyme ProAct (DSM, Parsippany, NJ, USA), 200 mg/kg (15,000 PRO/kg feed); Phy+Pro, combination of phytase and protease; Phy+Xyl, combination of phytase and xylanase; Xyl+Pro, combination of xylanase and protease; Phy+Xyl+Pro, combination of phytase, xylanase, and protease.

a–d Within a row, means without a common superscript letter differ (p<0.05).

**Table 4:** Apparent total tract digestibility (ATTD) of nutrients, and nitrogen retention (NR) of soybean meal in pigs^1)^ fed soybean meal diets with and without selected feed enzymes supplemented individually or combined

Item^2)^	Treatment^3)^								SEM	p value

	CON	Phy	Pro	Xyl	Phy+Pro	Phy+Xyl	Xyl+Pro	Phy+Xyl+Pro		
Soybean meal diet										
ATTD of DM (%)	90.3^d^	92.1^abcd^	93.8^abc^	91.7^bcd^	94.4^ab^	94.6^a^	90.1^d^	91.1^cd^	1.1	0.001
DE (kcal/kg)	3,402^b^	3,419^ab^	3,438^a^	3,415^ab^	3,436^a^	3,443^a^	3,397^b^	3,405^ab^	14	0.030
ME (kcal/kg)	3,296	3,291	3,323	3,310	3,347	3,336	3,291	3,298	20	0.240
ATTD of N (%)	88.2^abcd^	88.0^bcd^	89.2^a^	87.7^cd^	89.0^ab^	88.8^abc^	87.3^d^	88.1^abcd^	0.6	0.021
NR (%)	74.3	67.4	66.2	74.5	75.2	74.1	70.8	74.6	3.2	0.162
ATTD of ash (%)	67.5^bc^	69.9^ab^	68.3^bc^	68.4^bc^	72.1^a^	71.9^a^	66.2^c^	69.0^abc^	1.5	0.007
ATTD of Ca (%)	73.5^bc^	77.5^a^	73.8^bc^	73.8^bc^	78.5^a^	77.9^a^	72.4^c^	76.8^ab^	1.5	0.002
ATTD of P (%)	70.0^c^	76.6^a^	69.7^c^	71.5^bc^	74.0^ab^	74.5^ab^	68.6^c^	70.2^bc^	2.7	0.003
Soybean meal										
ATTD of DM (%)	92.4^d^	94.2^abcd^	95.9^abc^	93.8^bcd^	96.6^ab^	96.8^a^	92.3^d^	93.3^cd^	1.1	0.001
DE (kcal/kg)	3,427^b^	3,425^b^	3,479^a^	3,440^ab^	3,477^a^	3,478^a^	3,421^b^	3,430^ab^	21	0.030
ME (kcal/kg)	3,304	3,268	3,344	3,317	3,376	3,351	3,296	3,306	29	0.108
ATTD of N (%)	88.6^abc^	87.7^c^	89.7^a^	87.9^bc^	89.5^a^	89.1^ab^	87.6^c^	88.4^abc^	0.6	0.010
NR (%)	74.9	66.0	68.2	74.5	76.3	74.3	70.8	75.2	3.4	0.165
ATTD of ash (%)	50.9^bc^	54.4^ab^	52.0^bc^	52.1^bc^	57.8^a^	57.4^a^	48.9^c^	53.2^abc^	2.2	0.007
ATTD of Ca (%)	36.2^bc^	45.9^a^	36.9^bc^	36.8^bc^	48.2^a^	46.6^a^	33.6^c^	44.2^ab^	3.7	0.002
ATTD of P (%)	55.5^B^	62.4^A^	54.3^B^	58.1^AB^	57.5^AB^	60.4^AB^	54.0^B^	53.3^B^	4.2	0.098

SEM, standard error of the mean; ATTD of DM, apparent total tract digestibility of dry matter; DE, digestible energy; ME, metabolizable energy; ATTD of N, apparent total tract digestibility of nitrogen; NR, nitrogen retention; ATTD of ash, apparent total tract digestibility of ash; ATTD of Ca, apparent total tract digestibility of calcium; ATTD of P, apparent total tract digestibility of phosphorus.

1) Each least squares mean represents 4×4 Latin square replicated pens; 16 pigs were used in this experiment.

2) The values were calculated as dry matter basis.

3) CON, control treatment with no enzyme supplementation; Phy, Ronozyme Hiphos (DSM, Parsippany, NJ, USA), 100 mg/kg (1,000 FTU/kg feed); Pro, Ronozyme WX (DSM, Parsippany, NJ, USA), 200 mg/kg (200 FXU/kg feed); Xyl, Ronozyme ProAct (DSM, Parsippany, NJ, USA), 200 mg/kg (15,000 PRO/kg feed); Phy+Pro, combination of phytase and protease; Phy+Xyl, combination of phytase and xylanase; Xyl+Pro, combination of xylanase and protease; Phy+Xyl+Pro, combination of phytase, xylanase, and protease.

a–d Within a row, means without a common superscript letter differ (p<0.05).

A,B Within a row, means without a common superscript letter differ (0.05≤p<0.10).

**Table 5:** Apparent total tract digestibility (ATTD) of nutrients, and nitrogen retention (NR) of dried distillers grains with solubles (DDGS) in pigs^1)^ fed DDGS diets with and without selected feed enzymes supplemented individually or combined

Item^2)^	Treatment^3)^								SEM	p-value

	CON	Phy	Pro	Xyl	Phy+Pro	Phy+Xyl	Xyl+Pro	Phy+Xyl+Pro		
DDGS diet										
ATTD of DM (%)	82.9^d^	83.4^cd^	85.0^a^	84.8^ab^	83.9^bc^	84.8^ab^	83.5^cd^	82.5^d^	0.4	<0.001
DE (kcal/kg)	3,162^bc^	3,178^bc^	3,251^a^	3,242^a^	3,185^b^	3,236^a^	3,182^b^	3,120^c^	20	<0.001
ME (kcal/kg)	3,082^c^	3,085^c^	3,155^a^	3,139^ab^	3,094^bc^	3,134^ab^	3,072^c^	3,014^d^	18	<0.001
ATTD of N (%)	79.8^d^	80.2^cd^	82.2^a^	81.7^ab^	80.5^bcd^	81.3^abc^	80.4^bcd^	79.0^d^	0.7	0.001
NR (%)	61.4^AB^	58.7^B^	63.6^A^	61.1^AB^	61.7^AB^	60.6^AB^	58.6^B^	58.3^B^	1.5	0.081
ATTD of ash (%)	67.7^c^	72.0^a^	71.6^a^	70.6^ab^	71.6^a^	72.1^a^	68.6^bc^	68.3^bc^	0.9	<0.001
ATTD of Ca (%)	80.9^c^	85.7^a^	83.4^ab^	85.6^a^	86.0^a^	85.7^a^	82.4^bc^	81.7^bc^	1.3	<0.001
ATTD of P (%)	67.8^d^	74.7^a^	71.4^bc^	71.2^bc^	73.4^ab^	74.0^ab^	67.9^d^	69.3^cd^	1.4	<0.001
DDGS										
ATTD of DM (%)	64.7^d^	66.3^cd^	71.0^a^	70.4^ab^	67.7^bc^	70.6^ab^	66.6^cd^	63.4^d^	1.2	<0.001
DE (kcal/kg)	2,855^bc^	2,894^bc^	3,075^a^	3,052^a^	2,912^b^	3,039^a^	2,906^b^	2,751^c^	49	<0.001
ME (kcal/kg)	2,707^cd^	2,739^cd^	2,931^a^	2,893^ab^	2,769^bc^	2,869^ab^	2,734^cd^	2,576^d^	49	<0.001
ATTD of N (%)	76.8^d^	77.5^cd^	80.9^a^	79.9^ab^	78.0^bcd^	79.4^abc^	77.8^bcd^	75.4^d^	1.1	0.001
NR (%)	55.7^AB^	51.1^B^	59.4^A^	55.2^AB^	56.1^AB^	54.3^AB^	50.9^B^	50.4^B^	2.5	0.081
ATTD of ash (%)	51.4^c^	57.6^a^	57.2^a^	56.0^ab^	57.0^a^	57.8^a^	52.8^bc^	52.1^bc^	1.3	<0.001
ATTD of Ca (%)	45.1^c^	59.0^a^	52.2^ab^	54.5^a^	59.6^a^	58.7^a^	49.5^bc^	47.2^bc^	3.8	<0.001
ATTD of P (%)	64.6^d^	71.0^a^	68.4^abc^	68.7^ab^	69.1^ab^	70.7^a^	65.0^cd^	65.3^bcd^	1.6	<0.001

SEM, standard error of the mean; ATTD of DM, apparent total tract digestibility of dry matter; DE, digestible energy; ME, metabolizable energy; ATTD of N, apparent total tract digestibility of nitrogen; NR, nitrogen retention; ATTD of ash, apparent total tract digestibility of ash; ATTD of Ca, apparent total tract digestibility of calcium; ATTD of P, apparent total tract digestibility of phosphorus.

1) Each least squares mean represents 4×4 Latin square replicated pens; 16 pigs were used in this experiment.

2) The values were calculated as dry matter basis.

3) CON, control treatment with no enzyme supplementation; Phy, Ronozyme Hiphos (DSM, Parsippany, NJ, USA), 100 mg/kg (1,000 FTU/kg feed); Pro, Ronozyme WX (DSM, Parsippany, NJ, USA), 200 mg/kg (200 FXU/kg feed); Xyl, Ronozyme ProAct (DSM, Parsippany, NJ, USA), 200 mg/kg (15,000 PRO/kg feed); Phy+Pro, combination of phytase and protease; Phy+Xyl, combination of phytase and xylanase; Xyl+Pro, combination of xylanase and protease; Phy+Xyl+Pro, combination of phytase, xylanase, and protease.

a–d Within a row, means without a common superscript letter differ (p<0.05).

A,B Within a row, means without a common superscript letter differ (0.05≤p<0.10).

## Data Availability

All data generated or analyzed during this study are available from the corresponding author upon reasonable request.
